# Overexpression of CYP3A4 in a COLO 205 Colon Cancer Stem Cell Model *in vitro*

**DOI:** 10.3390/cancers3011467

**Published:** 2011-03-22

**Authors:** Ulrike Olszewski, Richard Liedauer, Christoph Ausch, Theresia Thalhammer, Gerhard Hamilton

**Affiliations:** 1 Ludwig Boltzmann Cluster of Translational Oncology, c/o Balderichgasse 26/13, A-1170 Vienna, Austria; E-Mail: ulrike.olszewski@toc.lbg.ac.at; 2 Department of Pathophysiology, Medical University of Vienna, Währinger Gürtel 18-20, A-1090, Vienna, Austria; E-Mails: richard.liedauer@meduniwien.ac.at (R.L.); theresia.thalhammer@meduniwien.ac.at (T.T.); 3 Department of Surgery, Danube Hospital, A-1220 Vienna, Austria; E-Mail: christoph.ausch@gmx.at

**Keywords:** colon cancer, cancer stem cells, CD133, CYP3A4, ALDH1A1

## Abstract

Cancer stem cells (CSCs) seem to constitute a subpopulation of tumor cells that escape from chemotherapy and cause recurrent disease. Low proliferation rates, protection in a stem cell niche and overexpression of drug resistance proteins are considered to confer chemoresistance. We established an *in vitro* colon CSC-like model using the COLO 205 cell line, which revealed transiently increased expression of CD133 when transferred to serum-free stem cell culture medium. Assessment of global gene expression of COLO 205 cells under these conditions identified a set of upregulated genes including cytochrome P450 3A4 (CYP3A4) and aldehyde dehydrogenase 1A1 (ALDH1A1), as confirmed by real-time qPCR. ALDH1A1 is a CSC marker for certain tumor entities and confers resistance to cyclophosphamide. CYP3A4 is expressed in liver and colon and its overexpression seems particularly relevant in colon cancer, since it inactivates irinotecan and other xenobiotics, such as taxols and vinca alkaloids. In conclusion, this COLO 205 model provides evidence for CD133 induction concomitant with overexpression of CYP3A4, which, together with ATP-binding cassette, subfamily G, member 2 (ABCG2) and others, may have a role in chemoresistant colon CSCs and a negative impact on disease-free survival in colon cancer patients.

## 1. Introduction

Advances have been made in the treatment of colon cancer, but despite apparent curative surgery and adjuvant chemotherapy, a significant number of patients succumb to recurrence of the disease [1]. In a new concept, limited effectiveness of standard anticancer therapies has been attributed to the existence of rare, highly drug resistant subpopulations of tumor cells termed cancer stem cells (CSCs) that are considered to be crucial for tumor initiation, development and impaired response to treatment [2,3]. A host of studies showed that CSC-enriched populations can be isolated and expanded from a variety of tumor entities, including glioblastomas, melanoma, breast, lung, ovarian and colon cancer [3-5]. Under serum-free conditions originally developed for embryonic or adult normal stem cells these cell populations preferentially grow as nonadherent, three-dimensional tumor spheres [6].

Although CSCs may represent a heterogeneous population, the glycoprotein prominin 1/CD133, an important marker of adult stem cells and CSCs, has been used for the isolation of several types of tumorigenic cells from brain tumors, as well as kidney, hepatocellular, colon, pancreatic and prostate carcinomas [2,3,7]. These studies demonstrated that the minor CD133^+^ subpopulation of cells reveals a much higher tumorigenic and clonogenic potential compared to CD133-depleted fractions or unsorted cells, and that CD133^+^ cells are profoundly resistant to chemo- and radiotherapy [3-5,8]. According to a conflicting study, CD133 expression in human colon is not restricted to the stem cells but ubiquitous in differentiated colonic epithelium as well as tumor cells expressing epithelial cell adhesion molecule (EpCAM) [9]. Both CD133^+^ and CD133^−^ metastatic tumor cell subpopulations formed colonospheres *in vitro* and were capable of tumorigenesis in a mouse xenotransplantation model. These data suggested that CD133 expression is not restricted to intestinal stem or cancer-initiating cells, respectively, and that CD133^+^ tumor cells might give rise to an even more aggressive CD133^-^ subset. Apart from this transient expression of CD133, it was determined that only a small fraction of CD133^+^ cells was able to initiate tumors [10]. It is obvious that more specific labels need to be identified to allow further characterization of CSCs; however, while superior markers are lacking, CD133 can be used in many cases to enrich these cells [11]. Although colon cancer specimens display a high variability in their patterns and levels of expression of some CSC markers, CD133 at least correlates with tumor aggressiveness and poorer clinical outcome [12-14].

Since isolated CD133^+^ cells are a mixture of real stem cells and early progenitor cells, selective conditions to enrich the genuine CSCs were developed. Cell sorting followed by cultivation in serum-free medium supplemented with suitable growth factors increased the proportion of CD133^+^ cells in association with their clonogenic capacity [6]. Genome-wide gene expression profiling of these cells showed increased transcription of genes related to anti-apoptosis, stemness, cell cycle/cell proliferation, transcription, DNA repair and many others [15]. The relatively quiescent CSCs seem to escape from chemotherapeutic regimens that typically target actively cycling cells and furthermore, they exhibit high expression levels of multidrug transporters, which likely results in a more efficient efflux of chemotherapeutic drugs and multidrug resistance [2,16,17]. Other pathways of drug detoxification like such effected by ALDH1 enzyme activity were reported [18]. Together with resistance to chemotherapy, CSCs are frequently refractory to standard radiotherapy due to increased expression of genes involved in DNA repair [8]. In good agreement with these findings, CRCs are enriched in residual tumors following chemotherapy and remain capable of rapidly regenerating tumors from which they were derived [19].

Reverse transcription-PCR (RT-PCR) analysis of CD133 expression in 32 colorectal cancer cell lines showed positive results in approximately two-thirds of the lines, including COLO 205 cells, and undetectable or low levels of CD133 expression in the remaining, possibly due to gene silencing by promoter hypermethylation of the CD133 CpG island [20]. The present study aimed to establish an *in vitro* colon CSC model using COLO 205 cells cultivated under serum-free stem cell conditions to search for increased expression of genes that are likely to be involved in chemoresistance of CSCs. Thereby, one highly expressed gene, newly identified by comparison of expression microarrays of normal and stem cell-like COLO 205 cells, namely CYP3A4, was further investigated in real-time qPCR and compared to the CSC marker ALDH1A1.

## 2. Results and Discussion

### 2.1. Serum-Free Stem Cell Culture of COLO 205 Cells and Expression of CD133

**Figure 1.**
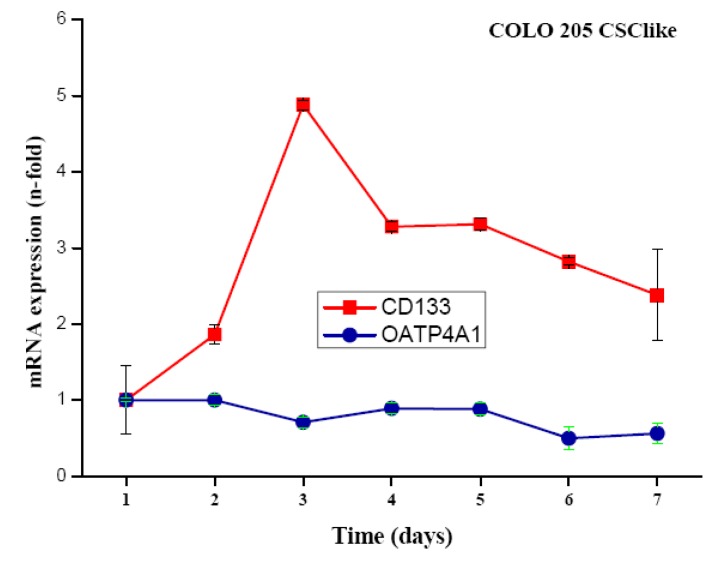
Expression of CD133 and OATP4A1 mRNA in serum-free stem cell medium. Time course of real-time qPCR of CD133 and OATP4A1 expression in COLO205 colon cancer cells cultivated in serum-free stem cell medium for the indicated period of time (mean ± SD). Expression of CD133 was significantly increased at days 2–7.

COLO 205 cells were transferred from standard serum-supplemented to serum-free stem cell medium containing the growth factors basic fibroblast growth factor (bFGF), epidermal growth factor (EGF), insulin-transferrin-selenite (ITS) solution and cultivated for up to seven days. Cells detached but did not form colonospheres. Expression of CD133 was measured in real-time qPCR at different time points and found to peak on day three after the transfer of the cells to stem cell medium, with expression levels declining slowly thereafter (Figure 1). To approve, the independent gene organic anion transporter polypeptide, family 4, member A1 (OATP4A1) showed no alteration of its expression under the same conditions.

### 2.2. Chemosensitivity of COLO 205 Cells Cultivated in Serum-Free Stem Cell Medium

**Figure 2.**
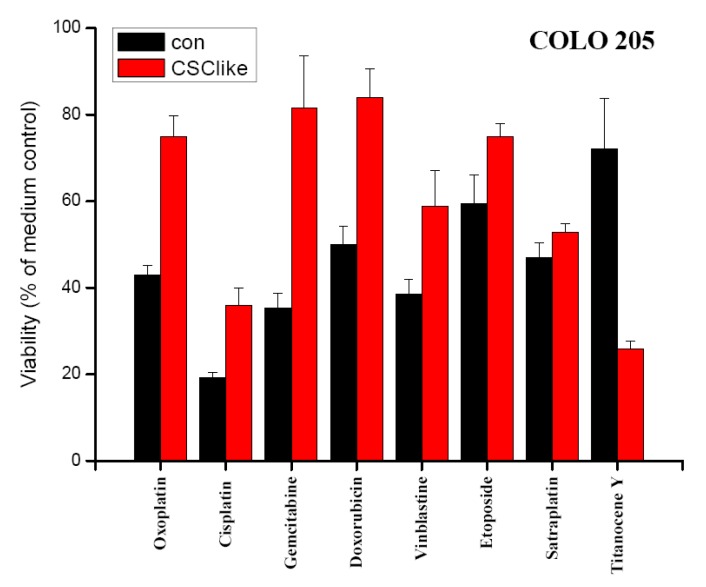
Comparison of chemosensitivities of COLO 205 cells precultivated in either standard medium or serum-free stem cell medium. Viability of COLO 205 colon cancer cells precultivated in either normal tissue culture (con) or stem cell medium (CSC-like) after exposure to a range of chemotherapeutics in normal tissue culture medium for four days *in vitro* (mean ± SD). All differences were statistically significant, except for satraplatin.

Chemosensitivity of COLO 205 cells cultivated in either standard tissue culture or serum-free stem cell medium *in vitro* was evaluated in MTT assays (Figure 2). Survival of cells was compared for a range of drugs used in definite concentrations near their respective IC_50_ values, with exception of cisplatin and titanocene Y. The IC_50_ values for COLO 205 cells had been calculated from dose-response-curves obtained in MTT assays using 6–8 two-fold dilution steps of the compounds in previous experiments (data not shown). Thus, drug concentrations deduced from these tests were 5 μg/mL for oxoplatin, 0.5 μg/mL for cisplatin, 1 μg/mL for gemcitabine, 2.5 μg/mL for doxorubicin, 1 μg/mL for vinblastine, 0.25 μg/mL for etoposide, 2.5 μg/mL (5 μM) for satraplatin and 25 μg/mL for titanocene Y, respectively. COLO 205 cells precultivated in serum-free stem cell medium revealed a significant increase in resistance to most cytotoxic drugs, except for satraplatin with comparable sensitivity for standard and stem cell-like cells and titanocene Y, which showed higher activity against the latter cells.

### 2.3. CYP3A4 and ALDH1A1 Expression in COLO 205 Cells Cultivated in Serum-free Stem Cell Medium

**Figure 3.**
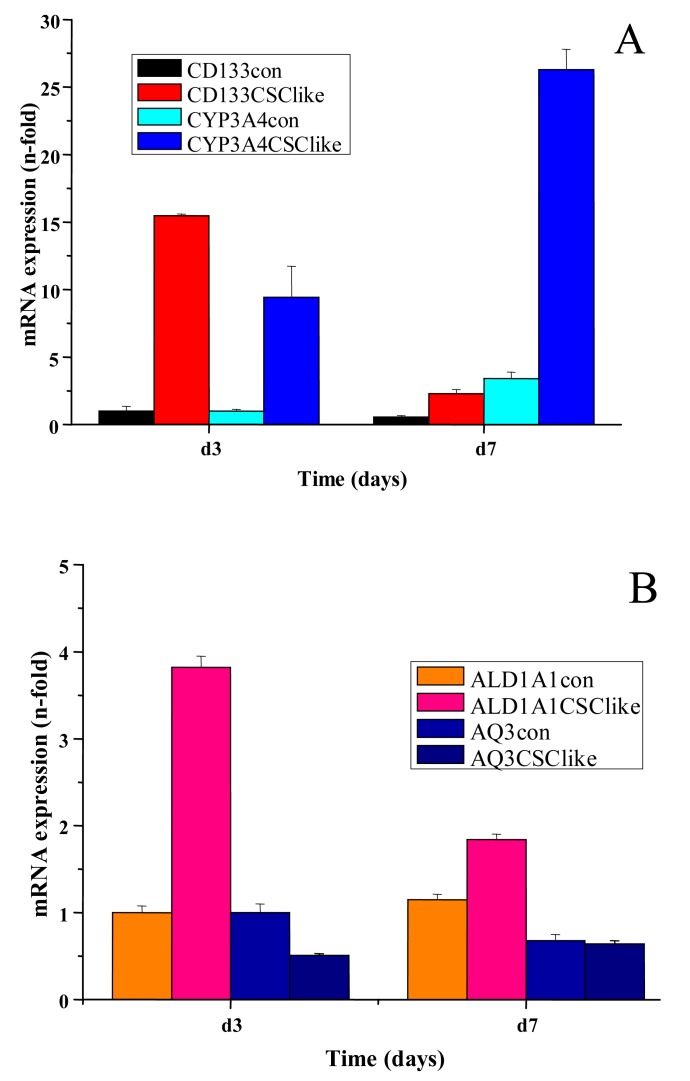
Assessment of gene expression levels of CD133, CYP3A4 (**A**) as well as ALDH1A1 and AQ3 (**B**) in COLO205 cells cultured in serum-free stem cell medium. Gene expression of the stem cell markers CD133 and ALDH1A1 in addition to CYP3A4 and AQ3 in COLO205 cells cultivated in either standard (con) or serum-free stem cell medium (CSClike) for three or seven days was measured by real-time qPCR (mean ± SD).

In another set of experiments, the changes in expression of CYP3A4, ALDH1A1 and aquaporin 3 (AQ3) in COLO205 were determined by real-time qPCR in cells after cultivation for three and seven days, in serum-free stem cell medium. After peaked expression of CD133 on day three, a marked increase in CYP3A4 expression was found on day seven (Figure 3A). Additionally, expression of the stem cell marker ALDH1A1 was moderately increased, whereas AQ3 showed a declining course between days three and seven (Figure 3B).

### 2.4. Discussion

Notable improvements in patient survival rates have been achieved for metastatic colorectal cancer in recent years, largely due to the availability of targeted molecular therapies in addition to the standard chemotherapeutic regimens; however, most patients still die of their disease [1]. Therefore, it is essential to understand the mechanisms of resistance as a first step in the development of approaches to prevent or reverse chemoresistance in patients receiving systemic treatment for metastatic colorectal cancer. According to a new concept, recurrent disease is caused by CSCs, a highly small but most chemoresistent subpopulation of tumor cells [2,21,22]. Colorectal CSCs can be identified using CD133 as detection marker and enriched by flow cytometric cell sorting [4,5]. CD133^+^ colorectal cancer cells were over 200-times more likely to initiate parental-like tumor growth in immunodeficient mice than CD133^−^ cells. Normal colonic mucosa contains a relatively low number of CD133^+^ cells compared with malignant mucosa. Since tumor-initiating CSCs are cycling quite slowly, they are less affected by cytotoxic therapies that target the transit-amplifying and differentiated cells, which form more than 99% of the tumor [22].

The CSC concept is still disputed for solid tumors, and there is growing evidence that both CD133^+^ and CD133^−^ populations can initiate tumors in distinct cases of colon cancer; however, patients with lower levels of CD133 exhibited longer relapse-free intervals and overall survival, regardless of adjuvant treatment and other clinical characteristics [9-11,23]. The clinical relevance of CD133 for metastasis of colorectal cancer was demonstrated by the detection of increased CD133 expression in patients with synchronous liver metastasis compared to those without dissemination to this organ [9,10]. It has been proposed that the failure to treat the cancer effectively may in part be due to the high resistance of CSC to chemotherapeutic agents [21]. CD133^+^ cells established from glioblastoma patients expressed higher levels of breast cancer resistance protein 1 (BCRP1) and O^6^-methylguanine-DNA methyltransferase (MGMT) mRNA, as well as increased mRNA levels of genes that inhibit apoptosis, for example of inhibitors of apoptosis (IAPs) [24,25]. These cells were resistant to chemotherapeutic agents like temozolomide, carboplatin, paclitaxel and etoposide, contrary to autologous CD133^-^ cells. Finally, CD133 expression was significantly elevated in recurrent glioblastoma tissue. The exclusion of Hoechst 33342 dye defines the pluripotent side population, where high drug efflux capacity correlates with the strong expression of the drug transporter protein BCRP1/ABCG2 [13,26]. CD133^+^ cells express higher levels of BCRP1, indicating an important role in the drug resistance of CD133^+^ cells. However, BCRP1 overexpressing tumor cells are not resistant to taxol and vincristine. Thus, the mechanisms of chemoresistance of CSCs remain to be fully investigated.

In our COLO205 CSC model transfer of the cells to serum-free stem cell medium resulted in transiently elevated expression of the stem cell marker CD133. The control gene OATP4A1, which was reported to show increased expression in the colon of patients with inflammatory bowel disease, remained unchanged upon the switch to serum-free medium conditions [27]. Control and COLO 205 stem cell-like cells were tested for their chemosensitivity to diverse cytotoxic agents, each applied at a definite concentration near the respective IC_50_ value in MTT assays. These IC_50_ concentrations for COLO 205 cells had been previously determined in MTT assays using the respective drugs in 6–8 twofold dilution steps (data not shown). As earlier reported cells with tumor stem cell characteristics exhibit higher resistance to chemotherapeutic drugs generally. In our experiments, satraplatin and titanocene Y were identified as compounds with similar or increased activity against CD133^+^ colon cancer cells [28,29]. Furthermore, these cells displayed significantly increased multidrug chemoresistance against the other cytotoxic drugs tested. In order to identify the genes related to increased resistance of these CSC-like COLO 205 cells, they were subjected to genome-wide gene expression analysis using microarrays (data not shown) [30]. Overexpression of CYP3A4, as revealed by this search, was confirmed by real-time qPCR and followed the transient increase in CD133 expression. Elevated translation of both CD133 and CYP3A4, as well as increases in their expression at the protein level, need to be confirmed. Additionally, higher mRNA expression of the colonic stem cell marker ALDH1A1 was detected in real-time qPCR [31]. Intracellular ALDH enzymes oxidize aldehydes to carboxylic acids and are involved in various catabolic processes, including ethanol and amine catabolism [32]. A subset of ALDH enzyme family members can degrade the bioactive metabolite of cyclophosphamide, namely 4-hydroxyperoxycyclophosphamide, but resistance to irinotecan appears to involve another mechanism [33].

Colon CSCs exhibit enhanced resistance to the standard chemotherapeutic irinotecan compared with their serum-cultured differentiated subcultures [34]. Escape of CSC subpopulations from irinotecan-mediated cell toxicity may be attributed to their relatively quiescent proliferative state [35]. Elevated expression of ABCG2 was reported for colon cancer tumor spheres and has also been anticipated to have a critical role in drug resistance of colon CSCs [26]. Irinotecan improves colorectal cancer treatment significantly with an efficacy superior to leucovorin-modulated 5-fluorouracil (5-FU), even in fluoropyrimidine-resistant neoplasms [36]. Preclinical studies proved the inhibition of topoisomerase I by 7-ethyl-10-hydroxycamptothecin (SN-38), the active metabolite of irinotecan and the possible synergistic interaction with other drugs effective against colorectal cancer, including 5-FU and oxaliplatin.

CYP3A4 is involved in the metabolism of a large number of drugs, and its overexpression in colonic CSC is expected to contribute to the resistance to anticancer therapy significantly [37]. Human tumors like colon, breast, lung, liver, kidney and prostate cancer are known to express CYP isoforms including members of the 3A and 1A subfamilies. CYP3A4 is involved in detoxification of irinotecan and SN-38 [37,38]. Other anticancer drugs affected include docetaxel, paclitaxel, gefitinib and erlotinib [39,40]. Sunitinib is a potent inhibitor of tyrosine kinase receptors used for the treatment of metastatic renal cell carcinoma and gastrointestinal stromal tumor patients who failed to respond to imatinib or were unable to tolerate it. Sunitinib is metabolized by CYP3A4 to the active metabolite SU12662 which is further converted to an inactive compound by the same enzyme [41]. On the contrary, CYP proteins act as reductases in hypoxic tumor regions and can accomplish tumor-specific prodrug activation, such as activation of alkylaminoanthraquinone N-oxide (AQ4N) by CYP3A [42].

## 3. Experimental Section

### 3.1. Chemicals, Cell Lines and Tissue Culture

Unless indicated otherwise, all chemicals were from Sigma-Aldrich (St. Louis, MO, USA). Oxoplatin and satraplatin were provided by Dr. Z. Salama (IPSS, Berlin, Germany) and titanocene Y by Dr. M. Tacke (University College, Dublin, Ireland). The COLO 205 cell line was obtained from the American Tissue Culture Collection (ATCC, Rockville, MD, USA) and cultivated in RPMI-1640 medium supplemented with 10% fetal bovine serum (FBS, Seromed, Berlin, Germany), 2 mm glutamine and antibiotics. Cells were subcultivated by trypsinization. For serum-free cell cultures, cells were washed with serum-free RPMI-1640 and transferred to serum-free RPMI-1640 medium supplemented with 2 mM glutamine, 4 ng/mL bFGF, 10 ng/mL EGF and insulin (10 μg/mL)/transferrin (5.5 μg/mL)/sodium selenite (5 ng/mL).

### 3.2. Chemosensitivity Tests

Mean IC50 values for the different compounds were obtained from dose-response curves. 1 × 10^4^ cells in 100 μL medium (RPMI-1640 / 10% FBS, 2 mM glutamine, antibiotics) were distributed to 96-well microtiter plates (Greiner, Kremsmuenster, Austria), and substances to be tested were added in a volume of another 100 μL. All compounds were serially diluted in 6–8 twofold steps in triplicate. The microtiter plates were incubated under tissue culture conditions (37 °C, 5% CO_2_, 95% humidity) for four days, and cell viability was measured using a modified MTT (3-(4,5-dimethylthiazol-2-yl)-2,5-diphenyl-tetrazolium bromide) assay (EZ4U, Biomedica, Vienna, Austria). Optical density was measured in a microplate reader at 450 nm with wells containing medium alone as reference. Values from wells containing cells and solvent alone were set to 100% proliferation.

### 3.3. mRNA Expression Analysis

Total RNA was isolated from cancer cell lines grown to subconfluency using the Trizol reagent according to the manufacturer's recommendations (Invitrogen, Lofer, Austria). Concentration, purity and integrity of RNA samples were determined on a Nanodrop ND-1000 spectrophotometer (Kisker-Biotech, Steinfurt, Germany) and by agarose gel electrophoresis. Reverse transcription of total RNA to cDNA (2 μg) was done with the high capacity cDNA reverse transcription kit (Applied Biosystems, Foster City, CA, USA). For the evaluation of mRNA expression of selected genes, real-time qPCR was done with TaqMan^®^ gene expression assays (Applied Biosystems). TaqMan^®^ probes were Hs00195682_m1 for CD133, Hs00946916_m1 for ALDH1A1, Hs00185020_m1 for AQ3 and Hs00604506_m1 for CYP3A4, respectively.

For normalization of real-time qPCR data, hypoxanthine phosphoribosyltransferase (HRPRT) (probe Hs01003267_m1) was chosen as reference gene in the analysis. The target gene amplification mixture contained 5 μL 2X TaqMan^®^ Gene Expression PCR Master Mix, 0.5 μL of the appropriate Gene Expression Assay, 10 ng template cDNA diluted in 2.5 μL nuclease-free water and 2 μL nuclease-free water. Thermal cycling conditions were as follows: 2 min at 50 °C, 10 min at 95 °C, 40 cycles of 15 s at 95 °C and 1 min at 60 °C. Fluorescence was measured with the ABI 7900HT Fast real-time qPCR system equipped with SDS 2.3 software (Applied Biosystems). All samples were amplified in duplicate. Results were imported into Microsoft Excel for further analysis, and relative cDNA amounts in the experimental samples were calculated as described by Hellemans *et al.* [43]. The difference (ΔC_T_) between the mean values in the duplicate samples of the target gene and those of HRPRT were calculated and the relative quantified value was expressed as 2^−ΔCT^.

### 3.4. Statistics

All the MTT and real-time qPCR assay results were analyzed by one way analysis of variance (ANOVA) followed by post-hoc (Tukey) tests using Winks (Texasoft; Broad St Cedar Hill, TX, USA). A level of P < 0.05 was considered statistically significant.

## 4. Conclusions

COLO 205 colon cancer cells exhibit transient CD133 expression in serum-free stem cell medium. CD133 is a label of colon CSCs linked to increased chemoresistance and represents a negative prognostic marker for colon cancer patients. The results of the present study demonstrate that, in addition to the drug-inactivating ALDH1A1, expression of CD133 in COLO 205 cells is followed by marked induction of CYP3A4, which plays a role in resistance to irinotecan, taxols, small-molecule EGFR inhibitors and other chemotherapeutics. In summary, the COLO 205 CSC-like model points to the involvement of CYP3A4 in chemoresistance of colon CSCs.

## References

[1] Dasari A., Messersmith W.A. (2010). New strategies in colorectal cancer: biomarkers of response to epidermal growth factor receptor monoclonal antibodies and potential therapeutic targets in phosphoinositide 3-kinase and mitogen-activated protein kinase pathways. Clin. Cancer Res..

[2] Pardal R., Clarke M.F., Morrison S.J. (2003). Applying the principles of stem cell biology to cancer. Nat. Rev. Cancer.

[3] Dalerba P., Cho R.W., Clarke M.F. (2007). Cancer stem cells: models and concepts. Annual Rev. Med..

[4] O'Brien C.A., Pollett A., Gallinger S., Dick J. (2007). A human colon cancer cell capable of initiating tumor growth in immunodeficient mice. Nature.

[5] Ricci-Vitiani L., Lombardi D.G., Pilozzi E., Biffoni M., Todaro M., Peschle C., De Maria R. (2007). Identification and expansion of human colon cancer-initiating cells. Nature.

[6] Fang D.D., Kim Y.J., Lee C.N., Aggarwal S., McKinnon K., Mesmer D., Norton J., Birse C.E., He T., Ruben S.M. (2010). Expansion of CD133(+) colon cancer cultures retaining stem cell properties to enable cancer stem cell target discovery. Br. J. Cancer..

[7] Shmelkov S.V., Clair R., Lyden D., Rafii S. (2005). AC133/CD133/Prominin-1. Int. J. Biochem. Cell Biol..

[8] Bao S., Wu Q., McLendon R.E., Hao Y., Shi Q., Hjelmelad A.B., Dewhirst M.W., Bigner D.D., Rich J.N. (2006). Glioma stem cells promote radioresistance by preferential activation of the DNA damage response. Nature.

[9] Shmelkov S.V., Butler J.M., Hooper A.T., Hormigo A., Kushner J., Milde T., St. Clair R., Baljevic M., White I., Jin D.K., Chadburn A., Murphy A.J., Valenzuela D.M., Gale N.W., Thurston G., Yancopoulos G.D., D'Angelica M., Kemeny N., Lyden D., Rafii S. (2008). CD133 expression is not restricted to stem cells, and both CD133+ and CD133- metastatic colon cancer cells initiate tumors. J. Clin. Invest..

[10] Yeung T.M., Mortensen N.J. (2009). Colorectal cancer stem cells. Dis. Colon Rectum..

[11] Wu Y., Wu P.Y. (2009). CD133 as a marker for cancer stem cells: progresses and concerns. Stem Cells Dev..

[12] Horst D., Kriegl L., Engel J., Kirchner T., Jung A. (2008). CD133 expression is an independent prognostic marker for low survival in colorectal cancer. Br. J. Cancer..

[13] Horst D., Scheel S.K., Liebmann S., Neumann J., Maatz S., Kirchner T., Jung A. (2009). The cancer stem cell marker CD133 has high prognostic impact but unknown functional relevance for the metastasis of human colon cancer. J. Pathol..

[14] Artells R., Moreno I., Díaz T., Martínez F., Gel B., Navarro A., Ibeas R., Moreno J., Monzó M. (2010). Tumour CD133 mRNA expression and clinical outcome in surgically resected colorectal cancer patients. Eur. J. Cancer.

[15] Botchkina I.L., Rowehl R.A., Rivadeneira D.E., Karpeh M.S, Crawford H., Dufour A., Ju J., Wang Y., Leyfman Y., Botchkina G.I. (2009). Phenotypic subpopulations of metastatic colon cancer stem cells: genomic analysis. Cancer Genomics Proteomics.

[16] Hirschmann-Jax C., Foster A.E., Wulf G.G., Nuchtern J.G., Jax T.W., Gobel U., Goodell M.A., Brenner M.K. (2004). A distinct “side population” of cells with high drug efflux capacity in human tumor cells. Proc. Natl. Acad. Sci. USA.

[17] Zhou J., Wang C.Y., Liu T., Wu B., Zhou F., Xiong J.X., Wu H.S., Tao J., Zhao G., Yang M., Gou S.M. (2008). Persistence of side population cells with high drug efflux capacity in pancreatic cancer. World J. Gastroenterol..

[18] Moreb J.S. (2008). Aldehyde dehydrogenase as a marker for stem cells. Curr. Stem Cell Res. Ther..

[19] Dylla S.J., Beviglia L., Park I.K., Chartier C., Raval J., Ngan L., Pickell K., Aguilar J., Lazetic S., Smith-Berdan S., Clarke M.F., Hoey T., Lewicki J., Gurney A.L. (2008). Colorectal cancer stem cells are enriched in xenogeneic tumors following chemotherapy. PLoS One..

[20] Jeon Y.K., Kim S.H., Choi S.H., Kim K.H., Yoo B.C., Ku J.L., Park J.G. (2010). Promoter hypermethylation and loss of CD133 gene expression in colorectal cancers. World J. Gastroenterol..

[21] Tang C., Ang B.T., Pervaiz S. (2007). Cancer stem cell: Target for anti-cancer therapy. FASEB J..

[22] Mimeault M., Hauke R., Mehra P.P., Batra S.K. (2007). Recent advances in cancer stem/progenitor cell research: Therapeutic implications for overcoming resistance to the most aggressive cancers. J. Cell Mol. Med..

[23] Gespach C. (2010). Stem cells and colon cancer: The questionable cancer stem cell hypothesis. Gastroenterol. Clin. Biol..

[24] Beier D., Hau P., Proescholdt M., Lohmeier A., Wischhusen J., Oefner P.J., Aigner L., Brawanski A., Bogdahn U., Beier C.P. (2007). CD133+ and CD133− glioblastoma-derived cancer stem cells show differential growth characteristics and molecular profiles. Cancer Res..

[25] Joo K.M., Kim S.Y., Jin X., Song S.Y., Kong D.S., Lee J.I., Jeon J.W., Kim M.H., Kang B.G., Jing Y., Jin J., Hong S.C., Park W.Y., Lee D.S., Kim H., Nam D.H. (2008). Clinical and biological implications of CD133-positive and CD133-negative cells in glioblastomas. Lab. Invest..

[26] Liu G., Yuan X., Zeng Z., Tunici P., Ng H., Abdulkadir I.R., Lu L., Irvin D., Black K.L., Yu J.S. (2006). Analysis of gene expression and chemoresistance of CD133+ cancer stem cells in glioblastoma. Mol. Cancer.

[27] Wojtal K.A., Eloranta J.J., Hruz P., Gutmann H., Drewe J., Staumann A., Beglinger C., Fried M., Kullak-Ublick G.A., Vavricka S.R. (2009). Changes in mRNA expression levels of solute carrier transporters in inflammatory bowel disease patients. Drug Metab. Dispos..

[28] Olszewski U., Hamilton G. (2010). A better platinum-based anticancer drug yet to come?. Anticancer Agents Med. Chem..

[29] Olszewski U., Hamilton G. (2010). Mechanisms of cytotoxicity of anticancer titanocenes. Anticancer Agents Med. Chem..

[30] Olszewski U., Zeillinger R., Geissler K., Hamilton G. (2010). Genome-wide gene expression analysis of chemoresistant pulmonary carcinoid cells. Lung Cancer: Targets and Therapy.

[31] Huang E.H., Hynes M.J., Zhang T., Ginestier C., Dontu G., Appelman H., Fields J.Z., Wicha M.S., Boman B.M. (2009). Aldehyde dehydrogenase 1 is a marker for normal and malignant human colonic stem cells (SC) and tracks SC overpopulation during colon tumorigenesis. Cancer Res..

[32] Moreb JS., Mohuczy D., Ostmark B., Zucali JR. (2007). RNAi-mediated knockdown of aldehyde dehydrogenase class-1A1 and class-3A1 is specific and reveals that each contributes equally to the resistance against 4-hydroperoxycyclophosphamide. Cancer Chemother Pharmacol..

[33] Sladek N.E., Kollander R., Sreerama L., Kiang D.T. (2002). Cellular levels of aldehyde dehydrogenases (ALDH1A1 and ALDH3A1) as predictors of therapeutic responses to cyclophosphamide-based chemotherapy of breast cancer: a retrospective study. Cancer Chemother. Pharmacol..

[34] Dylla S.J., Beviglia L., Park I.K., Chartier C., Raval J., Ngan L., Pickell K., Aguilar J., Lazetic S., Smith-Berdan S. (2008). Colorectal cancer stem cells are enriched in xenogeneic tumors following chemotherapy. PLoS One.

[35] Dallas N.A., Xia L., Fan F., Gray M.J., Gaur P., van Buren G., Samuel S., Kim M.P., Lim S.J., Ellis L.M. (2009). Chemoresistant colorectal cancer cells, the cancer stem cell phenotype, and increased sensitivity to insulin-like growth factor-I receptor inhibition. Cancer Res..

[36] Di Paolo A., Bocci G., Danesi R., Del Tacca M. (2006). Clinical pharmacokinetics of irinotecan-based chemotherapy in colorectal cancer patients. Curr. Clin.Pharmacol..

[37] Liu Y.T., Hao H.P., Liu C.X., Wang G.J., Xie H.G. (2007). Drugs as CYP3A probes, inducers, and inhibitors. Drug Metab. Rev..

[38] Candeil L., Gourdier I., Peyron D., Vezzio N., Copois V., Bibeau F., Orsetti B., Scheffer G.L., Ychou M., Khan Q.A., Pommier Y., Pau B., Martineau P., Del Rio M. (2004). ABCG2 overexpression in colon cancer cells resistant to SN38 and in irinotecan-treated metastases. Int. J. Cancer.

[39] Clarke S.J., Rivory L.P. (1999). Clinical pharmacokinetics of docetaxel. Clin. Pharmacokinet..

[40] Dimitroulakos J., Lorimer I.A., Goss G. (2006). Strategies to enhance epidermal growth factor inhibition: targeting the mevalonate pathway. Clin. Cancer Res..

[41] Adams V.R., Leggas M. (2007). Sunitinib malate for the treatment of metastatic renal cell carcinoma and gastrointestinal stromal tumors. Clin. Ther..

[42] Patterson L.H., McKeown S.R., Robson T., Gallagher R., Raleigh S.M., Orr S. (1999). Antitumour prodrug development using cytochrome P450 (CYP) mediated activation. Anticancer Drug Des..

[43] Jan Hellemans J., Mortier G., De Paepe A., Speleman F., Vandesompele J. (2007). qBase relative quantification framework and software for management and automated analysis of real-time quantitative PCR data. Genome Biol..

